# VIProDesign: Viral Protein Panel Design for Highly Variable Viruses to Evaluate Immune Responses and Identify Broadly Neutralizing Antibodies

**DOI:** 10.1101/2025.05.21.654924

**Published:** 2025-06-12

**Authors:** Tatsiana Bylund, Mohammed El Anbari, Andrew J Schaub, Tongqing Zhou, Reda Rawi

**Affiliations:** Vaccine Research Center, National Institute of Allergy and Infectious Diseases, National Institutes of Health, Bethesda, MD 20892, USA

**Keywords:** virus panels, Betacoronavirus, HIV-1, Influenza, Norovirus, Lassa

## Abstract

**Motivation:**

Highly mutable viruses continuously evolve, with some posing major pandemic risks. However, standardized neutralization assays and up-to-date viral panels are often lacking, limiting evaluation of immunogens and identification of broadly neutralizing antibodies. Closing these gaps is essential for guiding effective countermeasure development.

**Results:**

In this study, we present Viral Protein Panel Design (VIProDesign), a computational tool for designing viral protein panels that address the high sequence diversity of rapidly evolving viruses. VIProDesign uses the Partitioning Around Medoids (PAM) algorithm to select representative strains and applies the elbow-point method based on cumulative Shannon entropy to balance diversity and panel size. We used VIProDesign to generate optimized panels for Betacoronavirus, human immunodeficiency virus-1 (HIV-1), Influenza virus, Norovirus, and Lassa virus. The tool also supports customizable panel sizes, making it suitable for both resource-limited contexts and early-stage research. This flexible approach streamlines viral panel design across diverse pathogens. Although VIProDesign was originally developed for viral proteins, its underlying framework is broadly applicable to the selection of representative protein panels across diverse taxa, including bacterial species, toxins, and other biologically relevant protein families.

## 1 Introduction

Many viruses with pandemic potential, including Betacoronaviruses, HIV-1, and Influenza, exhibit substantial genetic variability and continuous evolution (Altman, et al., 2019; Bowen, et al., 2000; Chhabra, et al., 2019; Forni, et al., 2020; Hemelaar, 2012; Markov, et al., 2023; Mugosa, et al., 2016; Sarker and Jahan, 2023; Singh and Yi, 2021; Xue and Bloom, 2020). However, standardized panels for evaluating broadly neutralizing antibodies (bNAbs) and immunogen responses remain largely unavailable for most of these viruses due to significant cost and time constraints. The use of diverse pseudovirus strains across different studies further hinders the comparability of experimental results across studies. For instance, a panel of 60 HIV-1 isolates (Brown, et al., 2005) included 10 strains from each of the following six clades: A, B, C, D, CRF01_AE, and CRF02_AG, selected primarily based on availability, without prior analysis of population-level sequence diversity. In contrast, our in-house panel of 208 HIV-1 pseudoviruses (Doria-Rose et al., 2012) encompasses strains sampled between 1983 and 2008, offering broader temporal and genetic representation. A global panel of twelve HIV-1 Env strains (deCamp, et al., 2014) was developed using lasso regression to capture serum neutralization observed in a larger panel of 219 viruses. Most recently, Mkhize et al. (2023) analyzed HIV-1 viruses from 218 clinical trial participants collected between 2016 and 2020, primarily representing clades B and C. Complementary studies by Wieczorek et al. (Wieczorek, et al., 2024; Wieczorek, et al., 2023) proposed two focused panels: one comprising 30 contemporary subtype B pseudoviruses, and another including 50 recent subtype G and 18 CRF02_AG pseudoviruses derived from 10 Nigerian individuals across three studies conducted between 2003 and the present. Improving the relevance and utility of viral panels requires the inclusion of both historical and current strains to capture the full breadth of genetic diversity. Similar challenges exist across other viral pathogens, where the absence of standardized, representative panels hinders cross-study comparisons (Arevalo et al., 2022; Boyoglu-Barnum et al., 2021; Leonard et al., 2024; Moin et al., 2022). Incorporating genetically diverse and temporally distributed strains would enable more accurate assessments of immune responses and facilitate the development of broadly effective interventions. Selecting such representative sequence sub-sets often relies on clustering methods, which typically require users to define parameters such as sequence identity thresholds, the number of clusters (Balaban, et al., 2019; Edgar, 2010; Fu, et al., 2012; Mirdita, et al., 2021; Pipes and Nielsen, 2022; Wright, 2024), or other parameters (James, et al., 2018), which often necessitates prior estimation and iterative parameter optimization. ALFATClust (Chiu and Ong, 2022) dynamically determines an optimal cut-off threshold for each cluster by simultaneously evaluating cluster separation and intra-cluster sequence similarity. SpCLUST (Matar, et al., 2019) employs a combination of Laplacian eigenmaps for dimensionality reduction and Gaussian mixture modeling and uses BIC to define number of clusters.

In this study, we introduce Viral Protein Panel Design (VIProDesign), an in-silico tool for constructing viral panels that capture the genetic diversity of highly variable pathogens while balancing strain coverage and panel size. VIProDesign also enables the generation of panels with user-defined sizes, making it particularly suitable for resource-limited settings or early-phase studies focused on candidate bNAb screening. Using this framework, we designed optimized panels for Betacoronavirus, HIV-1, Influenza virus, Lassa virus, and Norovirus. To demonstrate its broader applicability beyond viral proteins, we applied VIProDesign to a dataset of three-finger toxins and successfully identified a representative panel. These results highlight VIProDesign as a scalable and reproducible framework for panel design, with broad utility across diverse applications, including vaccine development, antivenom generation, and therapeutic discovery.

## 2 Materials and methods

### 2.1 Data

All sequences analyzed in this study were obtained from the GenBank database between October 2022 and February 2025.

Specific search queries were constructed for each viral protein dataset:

#### Betacoronavirus:

(‘Betacoronavirus’[Organism] OR betacoronavirus[All Fields]) AND spike[All Fields] AND glycoprotein[All Fields]

#### Influenza:

(“Homo sapiens”[Organism] OR Human[All Fields]) AND Influenza[All Fields] AND HA[All Fields]

#### Norovirus:

(“Norovirus”[Organism] OR norovirus[All Fields]) AND (“Homo sapiens”[Organism] OR human[All Fields]) AND VP1[All Fields] NOT partial[All Fields]

#### Lassa Virus:

(“Mammarenavirus lassaense”[Organism] OR Mammarenavirus lassaense[All Fields]) AND glycoprotein[All Fields]

#### HIV-1:

Sequences were sourced from the HIV-1 Sequence Compendium 2021, curated by Los Alamos National Laboratory (Linchangco et al., 2021).

#### Three-finger toxins:

Data were retrieved from UniProt (https://www.uniprot.org) using the query: (“three finger toxin” OR “3FTx”) AND keyword:”Toxin [KW-0800]”, with sequences filtered by length (60–100 amino acids). All datasets used in this study are publicly available via Zenodo (https://doi.org/10.5281/zenodo.15389017).

### 2.2 Preprocessing

Sequences with non-standard amino acids including ‘X’ or partial sequences were excluded. Sequence redundancy was minimized using CD-HIT (Li and Godzik, 2006) at a 99% identity threshold. Outliers were detected and removed using the Density-Based Spatial Clustering of Applications with Noise (DBSCAN) algorithm. The optimal value of ε for DBSCAN for determining outliers was defined as the elbow of k-distance graph (Kumar, 2024). However, for datasets with limited sequences, outlier detection was omitted to preserve biologically relevant diversity.

### 2.3 Clustering

The preprocessed sequences were clustered over a specified range of k values using the Partitioning Around Medoids (PAM) algorithm (Kaufman, 1990), which selects a medoid, an actual data point that represents the central element of each cluster. Panels of sequences were constructed for each value of k, using the k medoids.

### 2.4 Entropy Analysis

Sequences within each constructed panel were aligned using the AlignSeqs function from the DECIPHER R package unless a custom alignment was provided. The Shannon entropy was calculated at each alignment position, and the cumulative sum of Shannon entropy across positions was computed to assess sequence diversity. The optimal panel size was identified using the elbow-point method, balancing maximum diversity with manageable panel size. Alternatively, the user can select a predefined panel size. The clustering_info reports the number of viruses in each cluster, along with key cluster statistics.

## 3 Results

**Figure 1:**
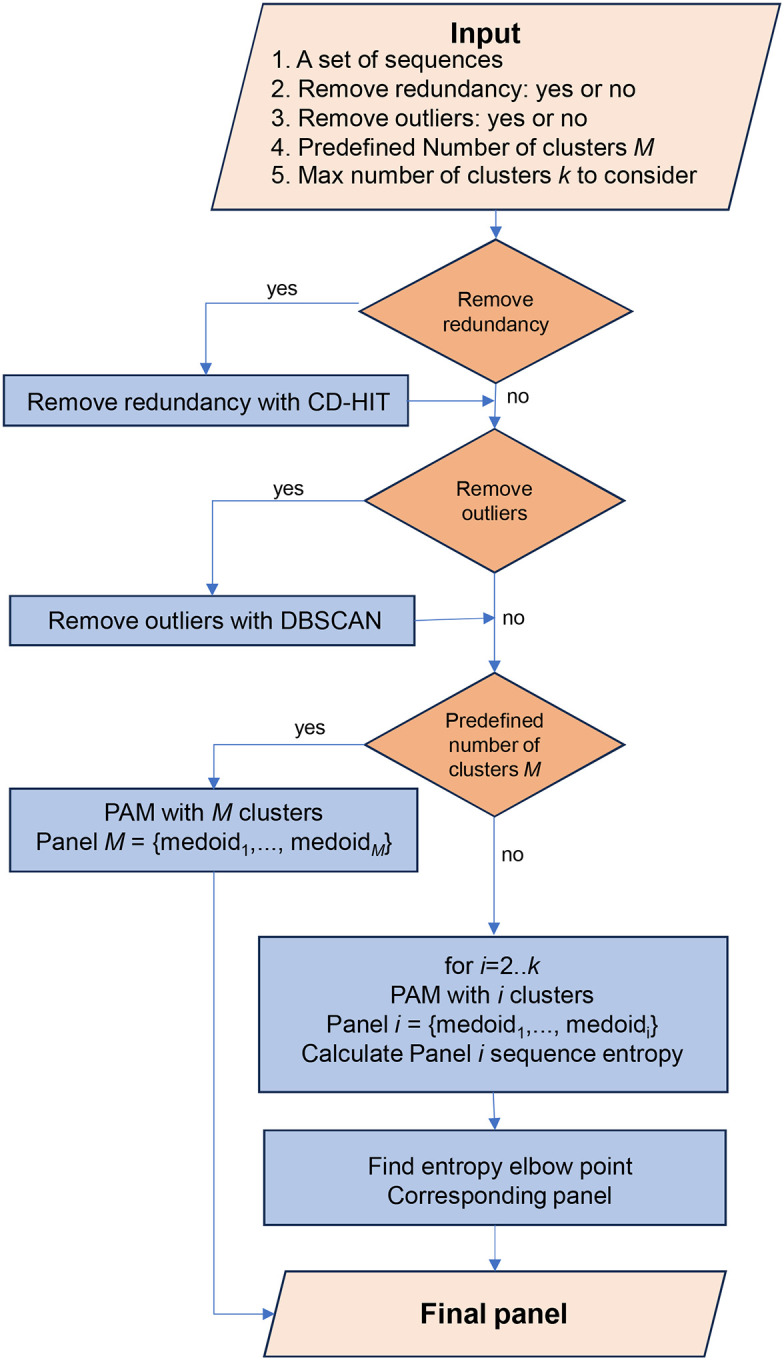
Flowchart of VIProDesign.

The design of comprehensive panels using VIProDesign follows the following procedure (Fig. 1). First, a set of sequences representing the target protein is assembled. The input data is then pre-processed by removing those containing unknown or ambiguous amino acid, typically represented by ‘X’. Next, sequence redundancy is removed by applying CD-HIT (Li and Godzik, 2006) with a 99% cutoff. The user also has the option to remove the outliers using DBSCAN algorithm (Kumar, 2024). If a predefined number of clusters, *M*, is specified, the PAM clustering algorithm is applied with *M* clusters, and the final panel consists of M cluster medoids. Alternatively, if a maximum number of clusters, *k*, is set instead, PAM clustering is performed for each value from two to k, and the final panel is selected at the elbow point of cumulative Shannon entropy.

**Figure 2:**
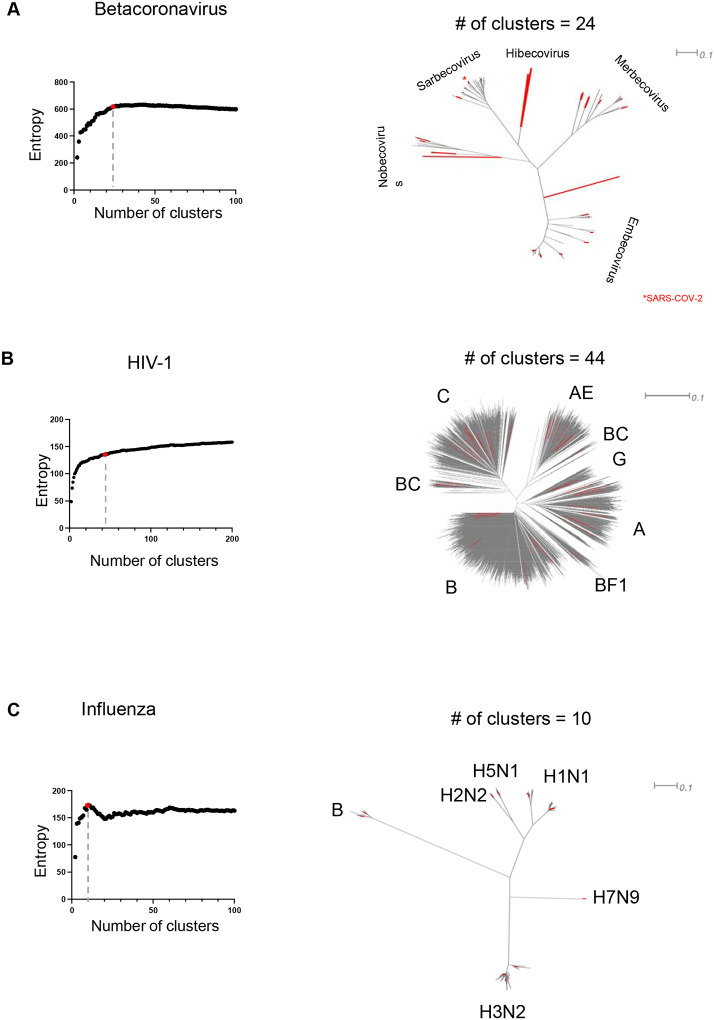
Panel designed for A) Betacoronavirus, B) HIV-1, C) Influenza. The sum of entropies is plotted against the number of clusters, with the elbow point highlighted in red in each graph. A phylogenetic tree representing the entire dataset is displayed, and the cluster centers corresponding to the elbow point are marked in red.

We applied VIProDesign to generate comprehensive panels for several highly variable viruses, including Betacoronavirus, HIV-1, Influenza virus, Norovirus, and Lassa virus. The proportion of outliers ranged from 3% for HIV-1 to 16% for Betacoronavirus (Supplementary Fig. 1). Due to the limited number of Betacoronavirus strains and higher proportion of outliers, we retained all Betacoronavirus sequences for the clustering step. Each viral dataset was clustered into k clusters using PAM, with *k* values ranging from 2 to 100, or up to 200 for HIV-1 due to its larger dataset size). A panel of *k* pseudoviruses was then designed, consisting of the *k* medoids from each cluster. The total Shannon entropy was calculated and plotted against the number of clusters for each *k* (Fig. 2). The optimal panel was selected at the elbow point of the entropy plot are named using the convention VIProDesign_*<virus name>_<number of strains>*.

The VIProDesign_Betacoronavirus_24 panel comprised 24 strains (Fig. 2a), including five Sarbecovirus, four Nobecovirus, two Hibecovirus, five Merbecovirus, and eight Embecovirus strains. VIProDesign_HIV-1_44 panel consists of 44 strains (Fig. 2b), representing all major clades: A, AE, B, BC, BF1, C, G, and D.

VIProDesign_Influenza_10 panel includes 10 strains: two H1N1 and three H3N2 strains, one H5N1, one H2N2, one H7N9, and one Influenza B strain. Similarly, comprehensive panels were designed for Norovirus (18 strains) and Lassa virus (10 strains) (Supplementary Figure 2, Table 1).

**Figure 3:**
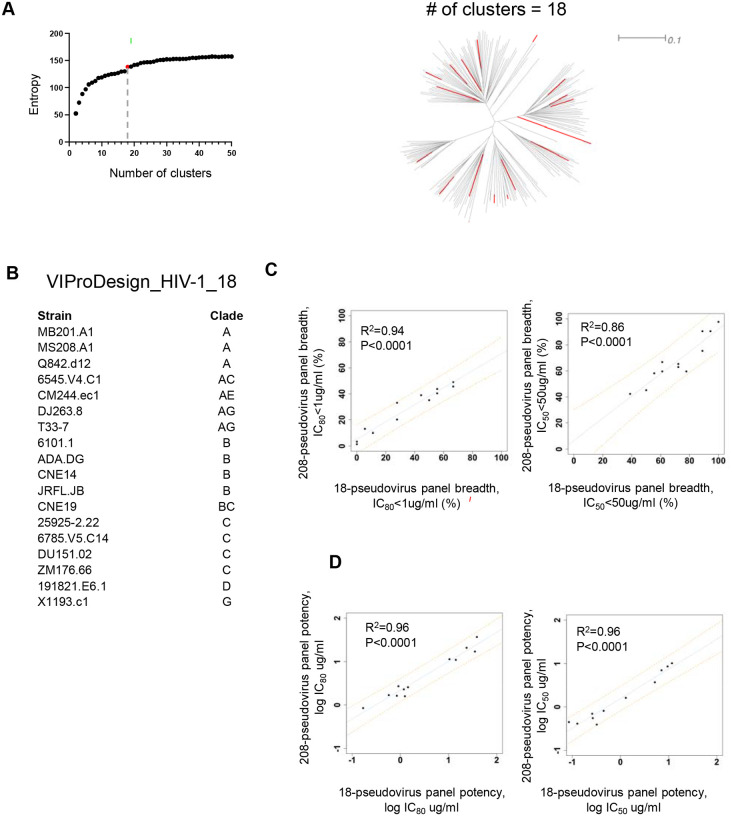
VIProDesign_HIV-1_18 panel designed based on commonly used HIV-1 208-pseudovirus set. A) The sum of entropies is plotted against the number of clusters. Elbow point = 18 is highlighted in red. The phylogenetic tree of 208 pseudoviruses is shown with 18 selected pseudoviruses highlighted in red. B) Designed HIV-1 panel. C) Linear regression of breadth (IC_80_<1 ug/ml and IC_50_<50 ug/ml) for 12 HIV-1 antibodies calculated based on 208-pseudovirus panel vs 18-pseudovirus panel. Predictive intervals are represented by orange dotted lines. D) Linear regression of potency for 12 independent HIV-1 antibodies calculated based on 208-pseudovirus panel vs 18-pseudovirus panel.

Our in-house HIV-1 208-pseudo virus panel for assessing bNAbs breadth and potency is often impractical during early-stage discovery, when hundreds of antibodies must be screened. To address this limitation, we used VIProDesign pipeline to derive a reduced panel from the 208-pseudovirus panel, resulting in VIProDesign_HIV-1_18 panel (Figure 3). The panel includes strains from all major clades – A, AG, AE, B, BC, C, D, and G. Despite its dramatically reduced size, VIProDesign_HIV-1_18 panel showed strong predictive power of antibody performance on the full 208-virus panel. Across 12 well-characterized bNAbs – PGT121, PGT151, PGT145, VRC01, CH235.12, CAP256V2LS, DH270_lb, HERH-c.01, 8ANC195, 35O22, 2F5, and 10E8 – VIProDesign_HIV-1_18 achieved high correlations with full-panel results (Fig. 3c, d), with R2 values of 0.94 for breadth (defined as IC80 < 1 μg/mL) and 0.96 for potency. These antibodies span diverse lineages and epitope specificities, and similar performance was observed in an expanded analysis of 53 bNAbs (Supplementary Table 2, Fig. 3). To accommodate resource-constrained settings, we further derived the VIProDesign_HIV-1_9 panel. While predictive performance is modestly reduced (R2 = 0.83 for breadth and 0.94 for potency), the 9-pseudovirus panel remains a practical and informative surrogate for full-panel assays (Supplementary Fig. 3).

Given that some existing HIV-1 panels focus exclusively on individual clades (Hraber et al., 2017; Wieczorek et al., 2023, 2024), we applied VIProDesign specifically to HIV-1 clades B and C (Supplementary Fig. 4). The resulting clade-specific panels are temporally and geographically diverse. The clade B panel VIProDesign_HIV-1_B_40 includes strains sampled between 1981 and 2018, while clade C panel VIProDesign_HIV-1_C_65 spans isolates collected from 1989 to 2018, with both panels incorporating strains from multiple countries.

Encouraged by the results obtained for the HIV-1 panels, we sought to generate a small Influenza panel that could accurately recapitulate antibody breadth and potency as measured against our in-house 55-virus panel (Creanga, et al., 2021). Applying VIProDesign on the 55-virus panel initially yielded a set of eight pseudoviruses, comprising two H1N1 and two H3N2 strains, one H2N2, one H5N1, one H7N9, and one H10N8. Given the biased distribution of H1N1 (n=23) and H3N2 (n=26) strains in the full panel, we subsequently applied the panel separately to H1N1 and H3N2 to ensure a representation of their diversity. This yielded six H1N1 and six H3N2 strains. We substituted the initial two H1N1 and two H3N2 with six H1N1 and six H3N2 strains, resulting in a final 16-virus. The optimized panel demonstrated strong predictive performance, achieving R^2^ values of 0.83 for breadth (IC_80_ < 1 μg/ml) and 0.94 for potency across 24 antibodies from the reference study (Supplementary Figure 4).

To further test the generalizability of VIProDesign beyond viruses, we applied it to a curated dataset of three-finger toxins, resulting in a representative panel of 20 toxin sequences (Figure 5). This demonstrates the framework’s potential utility for designing protein panels in toxinology and other non-viral applications.

## 4 Discussion

Many viruses, including Betacoronavirus, HIV-1, and Influenza virus, remain significant public health threats due to their high variability and continuous evolution. When evaluating potential immunogens and broadly neutralizing antibodies, it is crucial to use a comprehensive virus panel that accurately reflects viral diversity and is highly representative. A common approach to capturing this diversity is clustering and selecting cluster representatives. However, many clustering algorithms require the number of clusters and other parameters to be specified. For example, ROBINEN clustering (Al Hasan, et al., 2009), which stands for ROBust INitialization based on inverse DENsity estimator, requires the number of clusters and MinPts (the number of nearest neighbors) as input parameters to identify dense regions during initialization. Since different viruses exhibit varying degrees of variation, selecting a single value for MinPts that works for all viruses is impractical. Our analysis has shown that ROBIN clustering is sensitive to outliers. Using DBSCAN for determining the number of clusters resulted in only four clusters for Lassa and HIV-1, which fails to capture their diversity. We also considered Affinity Propagation clustering (Frey and Dueck, 2007). Similar to DBSCAN, it identifies clusters based on density, but it determines the number of clusters in an unsupervised manner. Affinity Propagation clustering produced 167 clusters for the HIV-1 dataset, a number too large to be practical for experimental validation. In comparison, ALFATClust identified a single cluster for Lassa virus and 424 clusters for HIV-1. SpCLUST, on the other hand, identified one cluster for HIV-1, two clusters for Norovirus, and three clusters for Lassa.

PAM clustering algorithm used in this study selects a medoid to represent the cluster. Although this algorithm is less sensitive to outliers than k-means, our examination showed that it is still affected by outliers. Since the goal of this research is to design a panel of representative viruses, we aim to remove strains that are farther from others using DBSCAN.

PAM requires *n*, the number of clusters, as a parameter for the algorithm. To determine the final value of n, we performed clustering for a range of n = 2 to 100. A panel of size n consisted of n medoids, which were selected after performing PAM clustering with n clusters. To evaluate the diversity of a panel, we calculated sum of normalized Shannon’s entropy for the panel. Entropy increases rapidly at first (see Figure 2, Supplementary Figure 2), but then either slows down (HIV-1, Lassa) or decreases (Betacoronavirus, Influenza, Norovirus). An increase in entropy reflects an increase in diversity of the panel sequences. Slight differences in entropy values for clusters of 50 and above reflect the fact that the sequences no longer contribute significantly to increasing diversity. Since we aimed to cover the diversity of the virus while maintaining a reasonable panel size, we chose the elbow point of Shannon’s entropy as the final panel. Another benefit of using PAM clustering is that it allows for the design of a predefined panel of a specified size.

The designed VIProDesign Betacoronavirus panel consists of 24 strains from five Betacoronavirus subgroups: Nobecovirus, Sarbecovirus, Hibecovirus, Merbecovirus, and Embecovirus. Not all Betacoronavirus subgroups infect humans, although some animal-borne infections are known to present risk to humans (Chen, et al., 2025; Frutos, et al., 2021). When the DBSCAN outlier detection algorithm was applied to the Betacoronavirus dataset (Supplementary Figure 1a), the Nobecovirus and Hibecovirus subgroups were eliminated. This suggests that outlier detection is not suitable in this case, due to the limited number of strains (n = 200) in the Betacoronavirus set.

The designed VIProDesign Influenza panel consists of 10 strains. It has representatives from Influenza B, H1N1, H2N2, H3N2, H5N1 and H7N9. The different shape of the entropy graph (Figure 2c) with distinct pick reflects the fact that there is little variation within each subtype. The average sequence identity between Influenza HA is 34% ± 39%, while sequence identity between H3N2 sequences is 93% ± 8%.

Since the commonly used HIV-1 208-pseudovirus panel is impractical for many immunogen and antibody evaluation studies, we aimed to design a more compact panel that would predict antibody breadth and potency (Figure 3). The designed VIProDesign HIV-1 18 panel is highly predictive of antibody breadth and potency on 208-pseudovirus panel (Figure 3c,d, Supplementary Figure 3a). Some experiments may require even smaller number of pseudoviruses due to limitations in experimental resources. Since VIProDesign enables the design of panels with a predefined size, we designed a VIProDesign_HIV-1_9 panel derived from the 208-pseudovirus panel (Supplementary Figure 3b). This panel includes pseudoviruses from all major clades (A, AE, AG, B, BC, C, D, and G) and is highly predictive of antibody breadth and potency (Supplementary Figure 3c). All pseudoviruses, except for 217–11, which is highly similar to T33–7, are also present in the 18-pseudovirus panel, which is very useful for follow-up experiments. An intermediate-sized panel may also be useful, and more accurate.

The designed VIProDesign HIV-1 comprehensive panel consists of 44 strains from all major clades. The sequence identity between HIV-1 strains ranges from 58% to 90%, with an average identity of 75% ± 3% between sequences.

When examining panels of different sizes, we observed that smaller panels are often subsets of larger ones. This reflects the fact that PAM selects a medoid from each cluster, and thus the centers of densely populated area are chosen. This is extremely beneficial, if a smaller panel is used in the initial steps of the experiments, and a larger size panel – in the later steps. A smaller panel will consist of representatives from the most densely populated regions, thereby reflecting the greater part of virus diversity. VIProDesign also outputs the number of viruses in each cluster, which could be useful to consider when selecting a panel.

**Figure 4:**
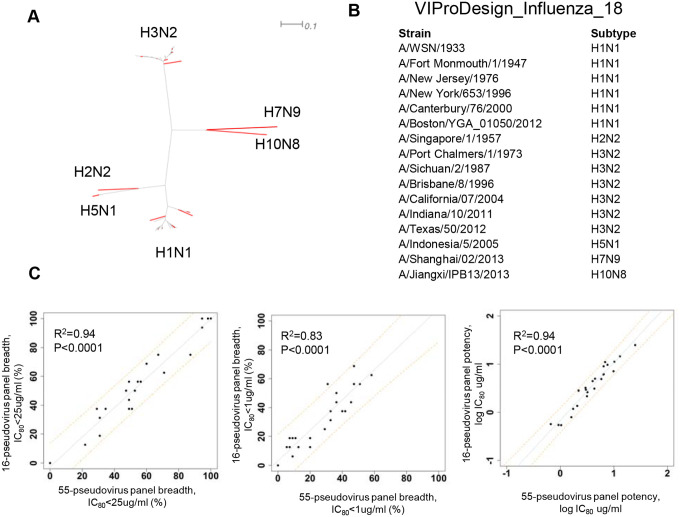
Influenza HA panel designed based on 55 Influenza panel. A) The phylogenetic tree of 55 strains is shown with 16 selected strains highlighted in red. B) Designed Influenza panel. C) Correlation of breadth (IC_80_<25 ug/m, IC_80_<1 ug/ml) and potency for 24 antibodies calculated based on 55-pseudovirus panel vs 16-pseudovirus panel. Predictive intervals are represented by orange dotted lines.

**Figure 5:**
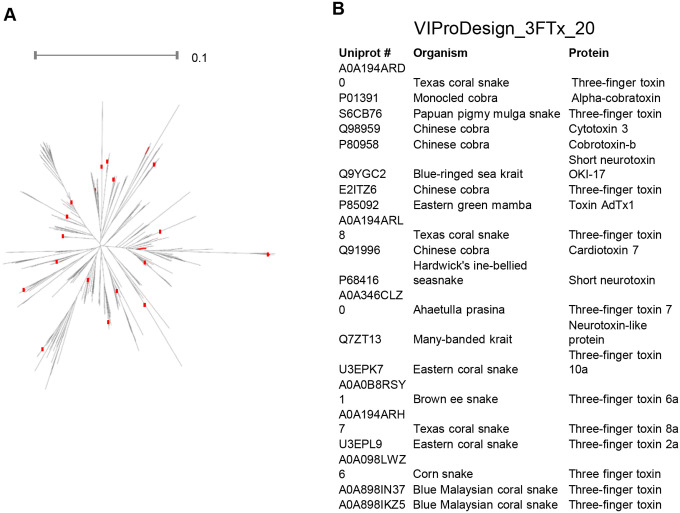
Three finger toxin panel. A) The phylogenetic tree of 560 three finger toxin sequences is shown with 20 selected strains highlighted in red. B) VIProDesign_3FTx_20 designed panel

The specifics of virus subgroup variation may require separate considerations when designing a comprehensive panel. Whereas HIV-1, Norovirus, and Lassa exhibit more uniform variability, as seen in the phylogenetic trees (Supplementary Figure 1), Influenza viruses exhibit a higher sequence identity difference between subtypes, approximately 40%, and a lower difference within a subtype, around 10%. When designing a comprehensive panel based on 55-virus panel (Creanga, et al., 2021) we needed to consider H1N1 and H3N2 separately, as the panel contained a large portion of viruses from both subtypes. After including six pseudoviruses from both H1N1 and H3N2, the final 16-pseudovirus panel proved to be highly predictive of antibody breadth and potency (Figure 4c).

Clade B and C are two of the major clades of HIV-1. Clade B is most prevalent in North America, Europe and Australia (Wieczorek, et al., 2023). Clade C is the most common lineage (Hraber, et al., 2017). It is interesting to note that the designed comprehensive panels included strains from a range of years, spanning from 1981 to 2018 (Supplementary Figure 4). It has been shown that HIV-1 virus evolves to become more resistant to broadly neutralizing antibodies (Mkhize, et al., 2023). When designing comprehensive panels, it is important to consider all available strains, as viruses are known to be reintroduced (Carter and Sanford, 2012). For example, the sequence identity between A/Alany/4835/1948//HA (Genbank: ABN59401.1) and A/Kiev/59/1979//HA (Genbank: AAA43172.1) is 98%, despite a 31-year difference between them. The sequence identity between B.US.06.04013440_5_B11.GU330809 and B.US.16.B207_CMV_CD69_G13.MT190767 is 91%, despite a 10-year difference, whereas sequence identity between two HIV-1 Env sequences from Brazil from the same year (B.BR.10.10BR_PE020.KT427739 and B.BR.10.10BR_PE006.KT427745) is 78%.

In this work, we developed VIProDesign, a novel approach for designing comprehensive virus panels for highly variable viruses. Our method utilizes Partitioning Around Medoids to select representative viruses and determines the panel size using the elbow point of Shannon entropy to maximize the diversity of the panel, while maintaining a reasonable panel size. This approach also allows for the creation of panels with a predefined size, making it especially valuable when smaller panels are needed due to resource constraints.

While VIProDesign was developed with viral proteins in mind, our application to three-finger toxins highlights the framework’s broader utility for generating compact, representative panels for other biologically diverse protein families. Three-finger toxins are a structurally conserved but functionally diverse class of snake venom proteins, and panel optimization in this space could be valuable for antivenom discovery, structure-function studies, and immunological profiling. This use case demonstrates that VIProDesign is not limited to virology and can be readily applied to other domains, including toxinology, parasitology, and bacterial pathogenesis – any context in which highly diverse protein repertoires complicate comprehensive screening or therapeutic development.

## Supplementary Material

Supplement 1

Supplement 2

1

## References

[R1] Al Hasan M., Robust partitional clustering by outlier and density insensitive seeding. Pattern Recogn Lett 2009;30(11):994–1002.

[R2] Altman M.O., Human Influenza A Virus Hemagglutinin Glycan Evolution Follows a Temporal Pattern to a Glycan Limit. mBio 2019;10(2). 10.1128/mBio.00204-19 PMC6445938 30940704

[R3] Balaban M., TreeCluster: Clustering biological sequences using phylogenetic trees. PLoS One 2019;14(8):e0221068. 31437182 10.1371/journal.pone.0221068 PMC6705769

[R4] Bowen M.D., Genetic diversity among Lassa virus strains. J Virol 2000;74(15):6992–7004. 10888638 10.1128/jvi.74.15.6992-7004.2000 PMC112216

[R5] Brown B.K., Biologic and genetic characterization of a panel of 60 human immunodeficiency virus type 1 isolates, representing clades A, B, C, D, CRF01_AE, and CRF02_AG, for the development and assessment of candidate vaccines. J Virol 2005;79(10):6089–6101. 15857994 10.1128/JVI.79.10.6089-6101.2005 PMC1091694

[R6] Carter R.W. and Sanford J.C. A new look at an old virus: patterns of mutation accumulation in the human H1N1 influenza virus since 1918. Theor Biol Med Model 2012;9:42. 23062055 10.1186/1742-4682-9-42 PMC3507676

[R7] Chen J., Bat-infecting merbecovirus HKU5-CoV lineage 2 can use human ACE2 as a cell entry receptor. Cell 2025. 10.1016/j.cell.2025.01.042 39970913

[R8] Chhabra P., Updated classification of norovirus genogroups and genotypes. J Gen Virol 2019;100(10):1393–1406. 31483239 10.1099/jgv.0.001318 PMC7011714

[R9] Chiu J.K.H. and Ong R.T. Clustering biological sequences with dynamic sequence similarity threshold. BMC Bioinformatics 2022;23(1):108. 35354426 10.1186/s12859-022-04643-9 PMC8969259

[R10] Creanga A., A comprehensive influenza reporter virus panel for high-throughput deep profiling of neutralizing antibodies. Nat Commun 2021;12(1):1722. 33741916 10.1038/s41467-021-21954-2 PMC7979723

[R11] deCamp A., Global panel of HIV-1 Env reference strains for standardized assessments of vaccine-elicited neutralizing antibodies. J Virol 2014;88(5):2489–2507. 24352443 10.1128/JVI.02853-13 PMC3958090

[R12] Edgar R.C. Search and clustering orders of magnitude faster than BLAST. Bioinformatics 2010;26(19):2460–2461. 20709691 10.1093/bioinformatics/btq461

[R13] Forni D., Cagliani R. and Sironi M. Recombination and Positive Selection Differentially Shaped the Diversity of Betacoronavirus Subgenera. Viruses 2020;12(11). 10.3390/v12111313 PMC7698105 33207802

[R14] Frutos R., Emergence of Bat-Related Betacoronaviruses: Hazard and Risks. Front Microbiol 2021;12:591535. 33790874 10.3389/fmicb.2021.591535 PMC8005542

[R15] Fu L., CD-HIT: accelerated for clustering the next-generation sequencing data. Bioinformatics 2012;28(23):3150–3152. 23060610 10.1093/bioinformatics/bts565 PMC3516142

[R16] Hemelaar J. The origin and diversity of the HIV-1 pandemic. Trends Mol Med 2012;18(3):182–192. 22240486 10.1016/j.molmed.2011.12.001

[R17] Hraber P., Panels of HIV-1 Subtype C Env Reference Strains for Standardized Neutralization Assessments. J Virol 2017;91(19). 10.1128/JVI.00991-17 PMC5599761 28747500

[R18] James B.T., Luczak B.B. and Girgis H.Z. MeShClust: an intelligent tool for clustering DNA sequences. Nucleic Acids Res 2018;46(14):e83. 29718317 10.1093/nar/gky315 PMC6101578

[R19] Kumar R. A Guide to the DBSCAN Clustering Algorithm. In. https://www.datacamp.com/tutorial/dbscan-clustering-algorithm; 2024.

[R20] Li W. and Godzik A. Cd-hit: a fast program for clustering and comparing large sets of protein or nucleotide sequences. Bioinformatics 2006;22(13):1658–1659. 16731699 10.1093/bioinformatics/btl158

[R21] Markov P.V., The evolution of SARS-CoV-2. Nat Rev Microbiol 2023;21(6):361–379. 37020110 10.1038/s41579-023-00878-2

[R22] Matar J., SpCLUST: Towards a fast and reliable clustering for potentially divergent biological sequences. Comput Biol Med 2019;114:103439. 31550555 10.1016/j.compbiomed.2019.103439

[R23] Mirdita M., Fast and sensitive taxonomic assignment to metagenomic contigs. Bioinformatics 2021;37(18):3029–3031. 33734313 10.1093/bioinformatics/btab184 PMC8479651

[R24] Mkhize N.N., Neutralization profiles of HIV-1 viruses from the VRC01 Antibody Mediated Prevention (AMP) trials. PLoS Pathog 2023;19(6):e1011469. 37384759 10.1371/journal.ppat.1011469 PMC10337935

[R25] Mugosa B., Genetic diversity of the haemagglutinin (HA) of human influenza a (H1N1) virus in montenegro: Focus on its origin and evolution. J Med Virol 2016;88(11):1905–1913. 27088433 10.1002/jmv.24552

[R26] Pipes L. and Nielsen R. AncestralClust: clustering of divergent nucleotide sequences by ancestral sequence reconstruction using phylogenetic trees. Bioinformatics 2022;38(3):663–670. 34668516 10.1093/bioinformatics/btab723 PMC8756197

[R27] Sarker M.S. and Jahan R. High Diversity and Transmission Dynamics of HIV-1 Non-C Subtypes in Bangladesh. Trop Med Infect Dis 2023;8(9). 10.3390/tropicalmed8090451 PMC10535170 37755912

[R28] Singh D. and Yi S.V. On the origin and evolution of SARS-CoV-2. Exp Mol Med 2021;53(4):537–547. 33864026 10.1038/s12276-021-00604-z PMC8050477

[R29] Wieczorek L., Differences in neutralizing antibody sensitivities and envelope characteristics indicate distinct antigenic properties of Nigerian HIV-1 subtype G and CRF02_AG. Virol J 2024;21(1):148. 38951814 10.1186/s12985-024-02394-y PMC11218331

[R30] Wieczorek L., Evolution of HIV-1 envelope towards reduced neutralization sensitivity, as demonstrated by contemporary HIV-1 subtype B from the United States. PLoS Pathog 2023;19(12):e1011780. 38055771 10.1371/journal.ppat.1011780 PMC10727358

[R31] Wright E. Accurately clustering biological sequences in linear time by relatedness sorting. Nat Commun 2024;15(1):3047. 38589369 10.1038/s41467-024-47371-9 PMC11001989

[R32] Xue K.S. and Bloom J.D. Linking influenza virus evolution within and between human hosts. Virus Evol 2020;6(1):veaa010. 32082616 10.1093/ve/veaa010 PMC7025719

