# Supplementary material for: VIProDesign: Viral Protein Panel Design for Highly Variable Viruses to Evaluate Immune Responses and Identify Broadly Neutralizing Antibodies
Source: bioRxiv. 2025 Jun 12:2025.05.21.654924. Preprint. [Version 2] doi: 10.1101/2025.05.21.654924 (PMC12190178; doi:10.1101/2025.05.21.654924)
Supplement: 1 [file NIHPP2025.05.21.654924V2-supplement-1.pdf]

bioRxiv preprint doi: https://doi.org/10.1101/2025.05.21.654924; this version posted June 12, 2025. The copyright holder for this preprint (which was not certified by peer review) is the author/funder. This article is a US Government work. It is not subject to copyright under 17 USC 105 and is also made available for use under a CC0 license.

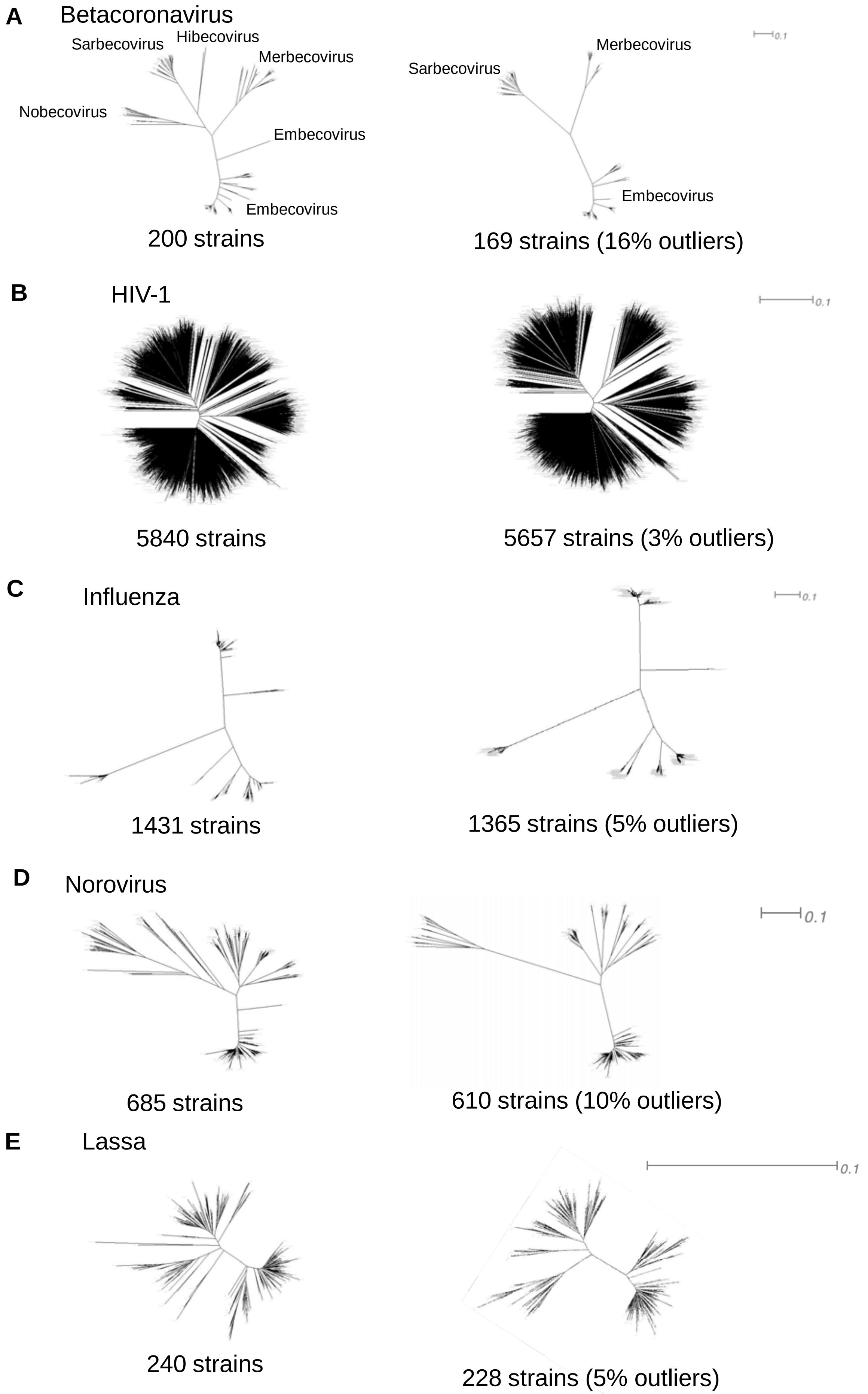

Supplementary Figure 1 Detecting outliers with DBSCAN. Phylogenetic trees of complete dataset and dataset with outliers removed are shown for A) Betacoronavirus, B) HIV-1, C) Influenza, D) Norovirus and E) Lassa.

bioRxiv preprint doi: https://doi.org/10.1101/2025.05.21.654924; this version posted June 12, 2025. The copyright holder for this preprint (which was not certified by peer review) is the author/funder. This article is a US Government work. It is not subject to copyright under 17 USC 105 and is also made available for use under a CC0 license.

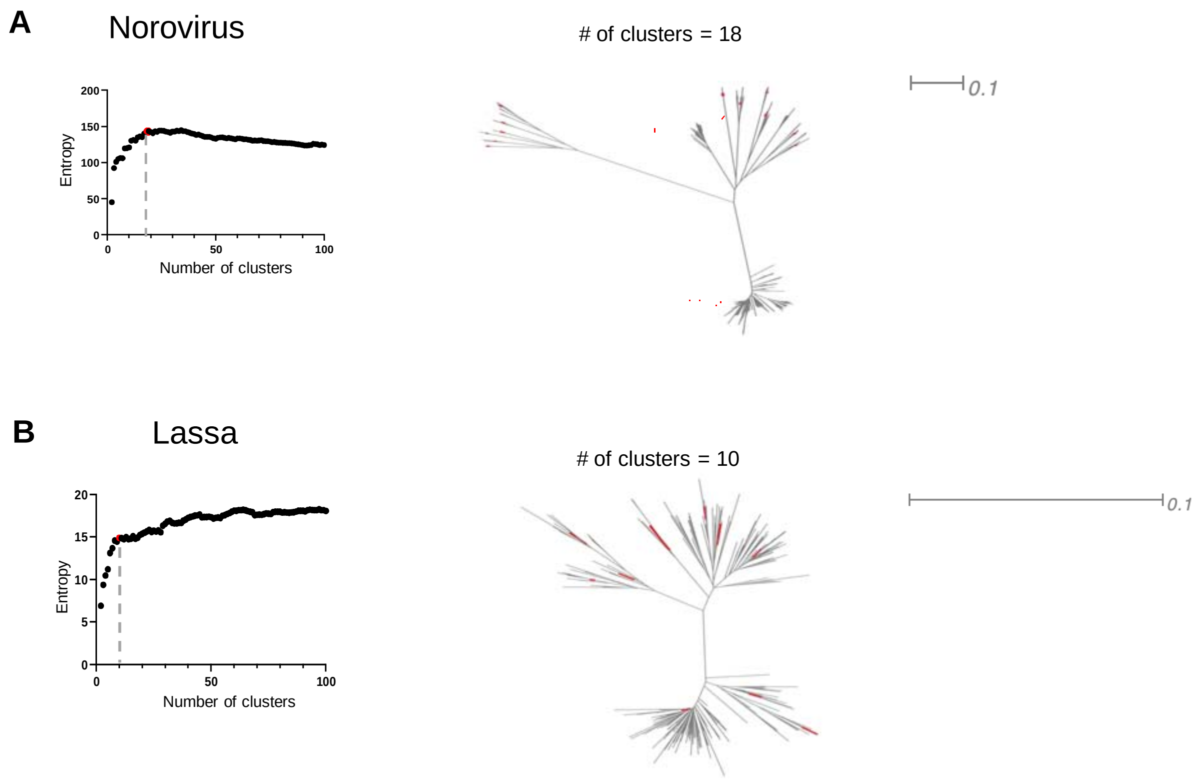

Supp Figure 2 Panel designed for A) Norovirus, B) Lassa. The sum of entropies is plotted against the number of clusters, with the elbow point highlighted in red in each graph. A phylogenetic tree representing the entire dataset is displayed, and the cluster centers corresponding to the elbow point are marked in red.

bioRxiv preprint doi: https://doi.org/10.1101/2025.05.21.654924; this version posted June 12, 2025. The copyright holder for this preprint (which was not certified by peer review) is the author/funder. This article is a US Government work. It is not subject to copyright under 17 USC 105 and is also made available for use under a CC0 license.

A

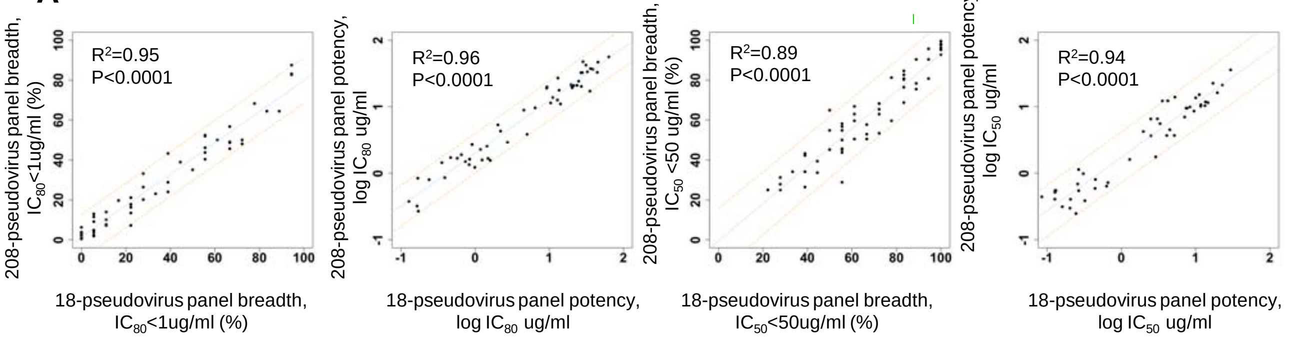

B

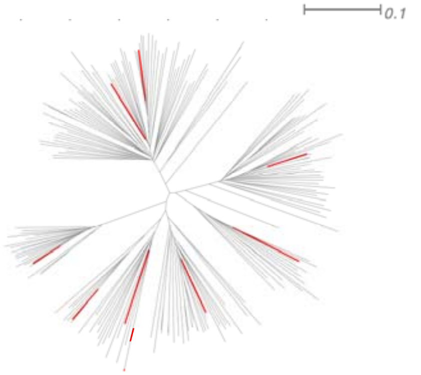

VIProDesign_HIV-1_9

Strain	Clade	# viruses in the cluster
Q842.d12	A	35
CM244.ec1	AE	23
271-11	AG	2
DJ263.8	AG	13
JRFL.JB	B	42
CNE19	BC	12
25925-2.22	C	59
191821.E6.1	D	15
X1193.c1	G	7

C

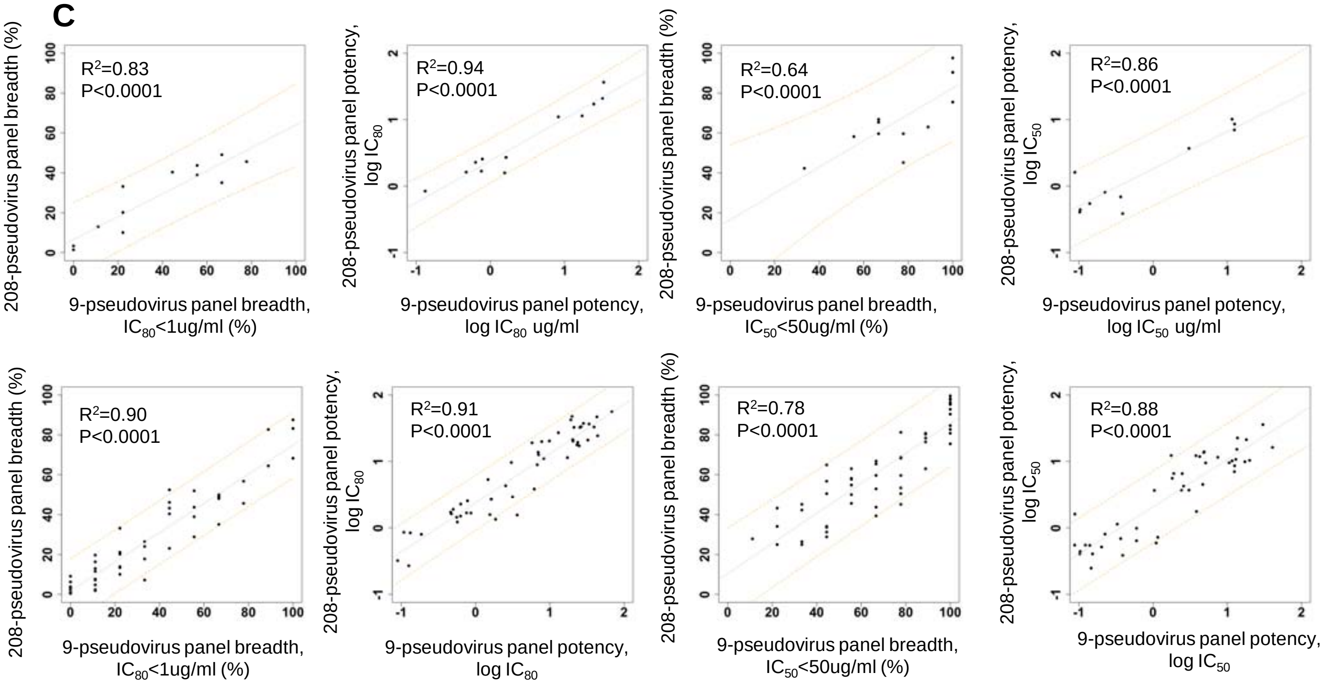

Supplientary Figure 3 HIV-1 panel designed based on commonly used HIV-1 208-pseudovirus set. **A**) Correlation of breadth and potency (IC_{50}<50 ug/ml) for 53 HIV-1 antibodies calculated based on 208-pseudovirus panel vs 18-pseudovirus panel. Predictive intervals are represented by orange dotted lines. **B**) The phylogenetic tree of 208 pseudoviruses is shown with 9 selected pseudoviruses highlighted in red. **C**) Correlation of breadth (IC_{80}< 1 ug/ml and IC_{50}<50 ug/ml) and potency for 12 independent HIV-1 antibodies and 53 HIV-1 antibodies calculated based on 208-pseudovirus panel vs 9-pseudovirus panel.

bioRxiv preprint doi: https://doi.org/10.1101/2025.05.21.654924; this version posted June 12, 2025. The copyright holder for this preprint (which was not certified by peer review) is the author/funder. This article is a US Government work. It is not subject to copyright under 17 USC 105 and is also made available for use under a CC0 license.

VIProDesign_HIV-1_B_40

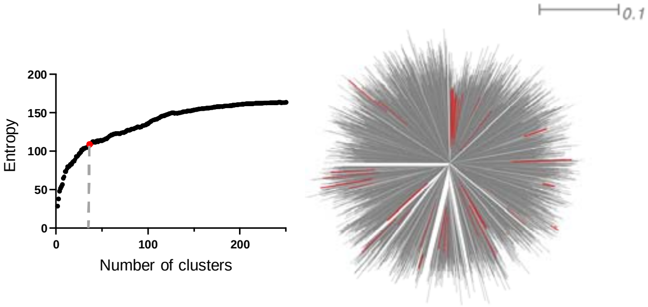

Strain	# in cluster
B.US.81.81NY3.AY247224	80
B.US.83.5157_83.AY835781	65
B.US.84.SC14C.U90935	226
B.US.86.SFMHS2.AF025750	492
B.US.86.YU_2.M93258	73
B.US.86.JRCSF.AY426125	75
B.FR.89.1197.KC699007	64
B.FR.89.749.KC699008	36
B.JP.89.31.AB588209	46
B.US.90.1580i.2.1.MT861860	152
B.US.91.SUMA_TF1.JN944928	67
B.KR.91.91OSG10.KF561442	56
B.TT.95.QH0864_M.AF277070	23
B.US.98.AC10.0.29.AY835446	7
B.ES.99.30_1_21.KC595159	136
B.JP.01.DR388.AB289590	4
B.ES.04.3227058_6Fc1.KC595162	80
B.US.07.ROCH4447_B17.HQ217509	62
B.CN.07.BJOX022000.e02.KM217995	13
B.US.x.DS_br_13.EU850430	69
B.CN.03.CHNHLJBSF03009c34.AY905493	73
B.CN.05.CNHLJBM05036.EU131803	31
B.BR.05.05BR1077.JN692460	3
B.KR.08.KR014.MT021905	5
B.ZA.09.21231341.HQ595755	2
B.JP.x.JRC03B.AB565496	10
B.US.10.DEMB10US003.KC473826	3
B.AR.10.DEMB10AR005.MN703127	3
B.BR.11.2011BRRJNEUT4.KX181894	3
B.US.12.2452_PBEM_13.KY778615	2
B.CN.12.555_0_8.KX693539	46
B.US.14.2531.KX505508	2
B.US.14.00720_PH259_w1_2_3half_17.MT307461	3
B.SE.15.102SE.MF373204	3
B.US.16.603_D14_D_P1_A11.MG196412	3
B.BR.16.14HI_2016.MK041573	3
B.BR.16.19HI_2016.MK041586	2
B.US.18.UNC_434_PL1_D2.MW062926	2
B.US.18.UNC_412_PL1_A10.MW062864	3
B.x.x.HypervirulentDutchHIV_10.MW689459	7

VIProDesign_HIV-1_C_65

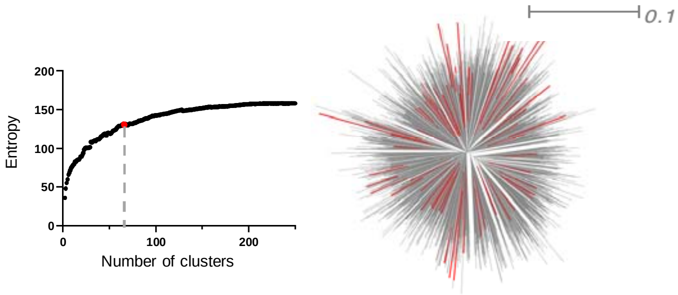

Strain	# in cluster
C.ZM.89.ZAM18.AB485647	59
C.DJ.91.DJ259A.L22940	10
C.BR.92.BR025_d.U52953	44
C.BE.93.VI882.HQ912708	26
C.MW.93.93MW_965.AY713413	38
C.IN.94.20635_4.JQ715368	48
C.BW.96.DEMC96BW002.KY658708	21
C.MW.96.C007_1E12_2B.HM638662	30
C.MW.96.C019_E1_3S.HM638911	55
C.MW.96.C047_H7_3B.KC894128	15
C.MW.96.C061_D6.KC894129	63
C.MW.96.C111_G3_3S.HM639196	2
C.MW.96.C120_3D3.KC863183	100
C.ZA.97.97ZA009.AY118166	32
C.IL.98.98IS002.AF286233	20
C.ZA.98.98ZADu104.AY529659	10
C.ZA.98.Du123_6.DQ411850	89
C.ZA.98.Du21.AY529661	36
C.ZA.99.99ZALT4.EU293444	27
C.BW.00.00BW20872.AF443104	11
C.BW.00.DEMC00BW010.KY658704	44
C.BW.00.DEMC00BW013.KY658705	31
C.ZA.00.1170MB.AY463225	43
C.ZA.00.1197MB.AY463234	24
C.ZA.00.J38MA.AY463228	22
C.ZM.02.02ZM110.AB254142	1
C.ZM.02.02ZM115.AB254148	33
C.ZM.02.2M.PB.2804.KY229352	3
C.ZM.02.3M.PB.351.KY229374	18
C.MW.03.0393_C3.KC894074	34
C.ZA.03.03ZAPS113MB2.DQ396365	8
C.ZA.03.03ZASK073B1.AY901970	2
C.ZA.03.03ZASK223B1.DQ093597	11
C.ZA.03.C.ZA.x.Du246_B3.KC863229	12
C.ZA.04.04ZAPS165MB1.DQ351221	29
C.ZA.04.SK140B1.AY901973	21
C.ZM.04.10B.PB.102.KY229612	2
C.ZM.04.11M.PL.803.KY229641	2
C.BW.05.3312_D6_2.KC154014	12
C.SE.05.011BI.MF373132	1
C.ZM.05.ZM416_200416_20.GU329473	1
C.ZA.06.CHV0008645_CAP269.2.00G7.FJ443911	1
C.TZ.06.CO6847V7.KX907425	3
C.MW.07.702010141_CH141.w12.KC156212	2
C.IN.07.NARI_VB52_J19.EU521727	55
C.ES.07.Read4_HIV_C.KX228820	6
C.ZA.07.704809221_1B3.KC894118	2
C.ZA.07.CH107_TF.MN202471	38
C.ZA.07.CHV0008639_705010015_H7.FJ443905	19
C.CY.08.CY219.JF683768	1
C.MW.08.702010432_CH432.w4.KC156218	2
C.NP.08.11NP084.KU341728	8
C.ZA.08.706010375g.MT395436	1
C.ZA.08.706010471_F4.HQ615967	25
C.ZA.08.SO032_A2.8_1.KF114894	82
C.ZM.09.Z3576M_18Apr09_PL_NFLG_SGA10.KR820326	2
C.MW.10.3036_CSF_Visit_1_amplicon19.KC187630	1
C.IN.11.NAB016.KY929361	2
C.IN.11.NAB063.KY929366	2
C.SE.11.063BI.MF373166	1
C.SE.12.068SE.MF373171	2
C.BW.13.mpp_00238_amp2.KR861328	2
C.MW.14.703010505_w323_3.MF353063	2
C.IN.15.330_15_A1.MK076687	1
C.IN.18.AIIMS743_74311A.MN703391	3

Supplementary Figure 4 HIV-1 clade B and C panels.
